# Clear Cell Adenocarcinoma of the Colon: A Case Report and Review of the Literature 

**DOI:** 10.1155/2014/905478

**Published:** 2014-03-04

**Authors:** Christian Daniel Barrera-Maldonado, Isidoro Wiener, Sue Sim

**Affiliations:** ^1^Department of Graduate Medical Education, Memorial Hermann Memorial City, 921 N Gessner Road, Houston, TX 77024, USA; ^2^General and Vascular Surgery, 902 Frostwood Suite 205, Prof. Building 1, Houston, TX 77024, USA; ^3^Department of Pathology, Memorial Hermann Memorial City, 921 N Gessner Road, Houston, TX 77024, USA

## Abstract

Clear cell adenocarcinoma of the colon has been described scarcely in the literature. It affects elderly men more commonly than women and usually appears in the left side of the colon. A Hispanic 41-year-old female came to the emergency room with abdominal pain, vomiting, and distension. Physical exam revealed generalized tenderness without peritoneal signs. Laboratory data was unremarkable. A CT scan showed an apple-core lesion in the distal colon. A flexible sigmoidoscopy revealed an obstructive mass that made further evaluation impossible. Exploratory surgery revealed a hard mass obstructing the descending colon, which was resected. Histopathology analysis with immunohistochemistry staining was positive for cytokeratin 20, cytokeratin 10, CDX2, and villin, while it was negative for cytokeratin 7, RCC, vimentin, and CD31. These results confirmed the clear cell variant of the adenocarcinoma. Clear cell adenocarcinomas usually arise from the kidneys and Müllerian organs. Immunohistochemistry is crucial for establishing the origin of these neoplastic cells. A cytokeratin 20+/7− with positive CDX2 is highly specific and sensitive for intestinal neoplastic origin. The main treatment has been surgery alone with moderately good results. More research and information about this malignancy is needed, especially in regard to prognosis and in order to provide the best treatment option.

## 1. Introduction

Clear cell adenocarcinoma of the colon is a well-recognized but very rare subtype of colorectal cancer. Its true incidence and prevalence are unknown. Fewer than 20 cases have been reported in the English literature. We report a case and also review the current literature about this subtype of neoplasia.

## 2. Case Report

A 41-year-old Hispanic female presented to the emergency room with abdominal pain, progressive distension, nausea and vomiting, and occasionally bloody stools for 2 months. She had an episode of melena one day prior to admission. Surgical history included a tubal ligation. She denied smoking, allergies, and medications as well as positive family history of cancer. On clinical examination, she had abdominal distension and mild generalized tenderness without peritoneal signs. The rectal examination was unremarkable. Hemoccult blood was negative. A contrast CT scan of the abdomen and pelvis revealed mild atelectasis in the lung bases, moderate distension of the gall bladder, and an apple core lesion in the distal descending colon measuring 3.4 cm long resulting in prominent dilation of the colon and small bowel proximal to this lesion. The colon distal to the lesion was not distended. There was also mild ascites. All laboratories including her basic metabolic panel, liver function tests, complete blood count, *α*-fetoprotein (AFP), and carcinoembryonic antigen (CEA) were within normal limits. She was started on pantoprazole and enoxaparin for deep venous thrombosis prophylaxis after an active bleeding was ruled out. A nasogastric tube was placed, but there was no fluid obtained. A flexible sigmoidoscopy was performed which revealed an obstructive mass that made evaluation of the colon above the mass impossible. Multiple biopsies were taken and displayed features that resembled adenocarcinoma of the colon.

She underwent surgical exploration. Intraoperative findings revealed an indurated, thick, obstructive lesion in the descending colon which was edematous but without perforation. A partial colectomy with end to end anastomosis was done.

**Figure 1:**
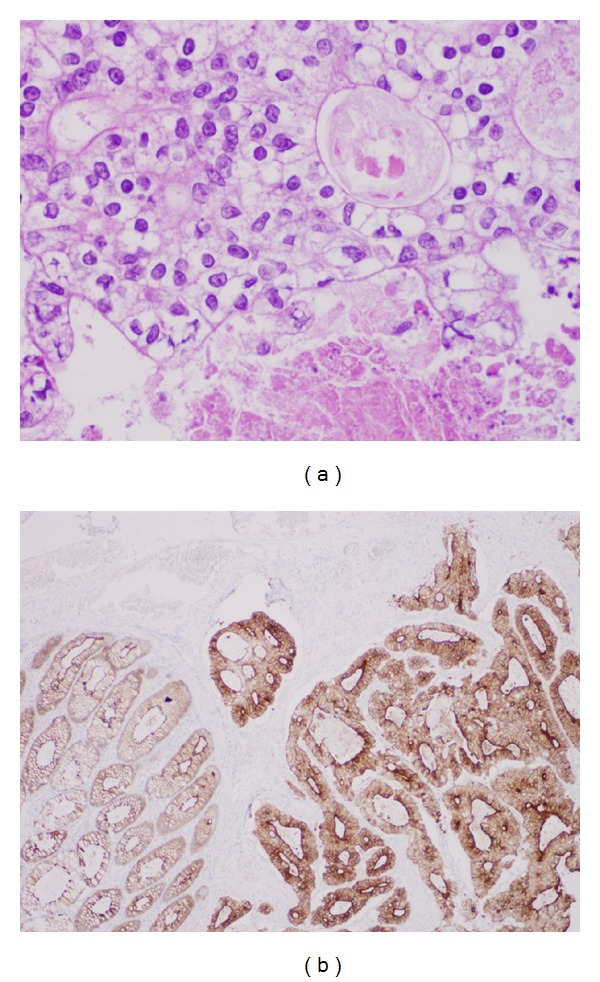
Histologic appearance of the clear cell adenocarcinoma of the colon. (a) H&E slide demonstrates rare variant of colonic adenocarcinoma with clear cell features. It shows gland-forming invasive tissue composed of columnar cells with unusually clear cytoplasm, prominent nucleolus, and central necrotic debris (400x). (b) Immunohistochemistry shows a strongly CDX2 positive staining of the malignant tissue (40x).

The surgery and postoperative course were uneventful. The histologic examination was consistent with colonic adenocarcinoma, with no perineural or vascular invasion noted. There was one positive lymph node out of 28 for metastatic tissue, and the specimen's margins were free of tumor. After the initial Hematoxylin and Eosin staining was performed, the clear cell variant was considered (Figure 1(a)). Subsequently, immunohistochemical stains, which were positive for cytokeratin (CK) 20, CK10, CDX2, and villin, combined with negative CK7, RCC, vimentin, and CD31 (Figure 1(b)), confirmed the clear cell subtype. These results are summarized in Table 1.

The tumor was staged American Joint Committee on Cancer (AJCC) IIIA, C, and C1 according to the Dukes and Astler-Coller staging systems, respectively. Tumor grade was moderately differentiated.

After the surgery, the patient was discharged on the fifth postoperative day in stable condition.

## 3. Discussion

Since first reported in 1964 by Hellstrom and Fisher [1], the clear cell adenocarcinoma of the colon (CCACC) has been described very few times. According to our searches in PubMed, only 14 cases have been reported in the English literature. Its true incidence and prevalence remain unknown. In a study published in 1999, out of 3,486 cases of colon cancer, only 3 (0.086%) were reported having clear cell variant [2–5]. There are no newer similar studies. The clear-aspect origin of these cells is still unknown; while some authors argue that the clear appearance of the cell comes from glycogen accumulation [6], some others failed to demonstrate this [3]. Most commonly, clear cell adenocarcinomas originate from the kidneys and Müllerian organs, such as the uterus, fallopian tubes, and ovaries [5]. Some cases of endometriotic dysplastic glands with clear cell changes in the colon have been reported, and they are known as endometriosis-associated intestinal tumors (EAIT) [5]. According to our review, males are most commonly affected than females, usually between the 6th and 7th decades of life, and the primary lesion most commonly involves the left colon [2–4, 7, 8]. Some authors have suggested and found a carcinogenic progression from adenoma to clear cell change adenoma and clear cell adenocarcinoma [3, 4, 8, 9]. However, because of the negative past history and the acute presentation of our patient, it was impossible to determine this sequence. Since there are few reports of this subtype of cancer with followups, prognosis cannot be adequately established.

According to current literature, one way to differentiate a primary intestinal neoplasia from a Müllerian neoplasia is with cytokeratins [3–5, 10–14]. Cytokeratins (CK) are composed of at least 20 structural proteins of epithelial cells and their patterns of expression vary depending on the location of the epithelium. CK20 is a type I keratin encoded by the gene KRT20 with a molecular weight of 46 kDa. CK7 is a type II keratin usually found in nonkeratinizing epithelia with a molecular weight of 54 kDa [4]. A positive CK20 and a negative CK7 would indicate an intestinal origin of the neoplasia with great accuracy because CK20 is usually expressed in the intestinal and gastric epithelium and the endocrine cells in the upper portions of the pyloric glands, whereas CK7 is usually found in the breast, lung, ovary, and urothelium [3–5, 11, 15]. CDX2 is another marker available for intestinal neoplasia [12–14]. It is a homeobox transcription factor in charge of development of intestinal epithelium, expressed in the small and large intestine and it correlates with intestinal metaplasia, dysplasia, and cancer. Because of this, although some studies argue that the loss of CDX2 is prevalent among dysplastic and malignant cells of gastric cancer and suggested the same rule for colonic tumors [13], newer studies contradict these findings and identify CDX2 antibody as a useful tumor marker specific for neoplasia of intestinal origin, although not as specific as CK7/CK20 [11, 12, 14]. Villin is a 9.5 kDa actin-binding protein expressed almost exclusively in the intestinal and renal epithelia. It has been well established as a useful aid in identifying metastatic gastrointestinal malignancies along with cytokeratins [12]. The clinical and histological characteristics of this patient's tumor were compatible with reported cases of CCACC.

There is no study that evaluates the invasive characteristics or tendencies of this type of tumor. It is interesting to note that there have been no reported cases of perineural invasion. Surgery has been the main treatment in the reported cases. Four cases reported metastatic tissue either at time of diagnosis or after resection of the primary tumor [5, 7, 10, 16]. The main affected organs were the liver and lungs. There are some cases in which adjuvant chemotherapy has been used, but there are no reports or studies addressing the usefulness, superiority, or inferiority of chemo- or radiotherapy [5]. Our patient had an excellent recovery and moved out of the country where she will have followups with an oncologist.

We can conclude that there are still some major aspects of CCACC that need to be further studied. An actual review of incidence, prevalence, prognosis, and survival is recommended. The clear cell origin of these tumors needs to be elucidated. Moreover, an adequate algorithm or plan for treatment must be elaborated.

## Figures and Tables

**Table 1:** Summary of the tumor's immunohistochemistry report.

Marker	Result
Cytokeratin 20	+
Cytokeratin 10	+
Vimentin	−
Villin	+
CDX2	+
Cytokeratin 7	−
RCC	−
CD31	−
